# Evaluation of emergency department performance – a systematic review on recommended performance and quality-in-care measures

**DOI:** 10.1186/1757-7241-21-62

**Published:** 2013-08-09

**Authors:** Christian Michel Sørup, Peter Jacobsen, Jakob Lundager Forberg

**Affiliations:** 1 DTU Management Engineering, Technical University of Denmark, Produktionstorvet, building 424, 2800, Kongens Lyngby, Denmark; 2 The Emergency Department, Nordsjællands Hospital, Dyrehavevej 29, 3400, Hillerød, Denmark

**Keywords:** Performance, Measures, Indicators, Emergency department, Quality improvement, Quality

## Abstract

**Background:**

Evaluation of emergency department (ED) performance remains a difficult task due to the lack of consensus on performance measures that reflects high quality, efficiency, and sustainability.

**Aim:**

To describe, map, and critically evaluate which performance measures that the published literature regard as being most relevant in assessing overall ED performance.

**Methods:**

Following the PRISMA guidelines, a systematic literature review of review articles reporting accentuated ED performance measures was conducted in the databases of PubMed, Cochrane Library, and Web of Science. Study eligibility criteria includes: 1) the main purpose was to discuss, analyse, or promote performance measures best reflecting ED performance, 2) the article was a review article, and 3) the article reported macro-level performance measures, thus reflecting an overall departmental performance level.

**Results:**

A number of articles addresses this study’s objective (n = 14 of 46 unique hits). Time intervals and patient-related measures were dominant in the identified performance measures in review articles from US, UK, Sweden and Canada. Length of stay (LOS), time between patient arrival to initial clinical assessment, and time between patient arrivals to admission were highlighted by the majority of articles. Concurrently, “patients left without being seen” (LWBS), unplanned re-attendance within a maximum of 72 hours, mortality/morbidity, and number of unintended incidents were the most highlighted performance measures that related directly to the patient. Performance measures related to employees were only stated in two of the 14 included articles.

**Conclusions:**

A total of 55 ED performance measures were identified. ED time intervals were the most recommended performance measures followed by patient centeredness and safety performance measures. ED employee related performance measures were rarely mentioned in the investigated literature. The study’s results allow for advancement towards improved performance measurement and standardised assessment across EDs.

## Background

In Europe, many EDs have undergone organisational changes [1,2]. Hospitals receiving acute patients are increasingly merged to larger organizations. Continuous high expertise in the EDs is promoted through the presence of relevant resources, medical specialties and experienced staff [2]. In Denmark, the new concept behind EDs consists of merging all acute admission units and observatory units into one joint ED. The rationale for re-structuring is first and foremost to cope with an increased amount of patients while securing delivery of high quality and efficiency, concurrently with decreased overall hospital capacity [3]. Promotion of interdisciplinary teamwork and earlier senior physician involvement are examples of means to deliver timely and high quality treatment to patients within the EDs, which is essential for early diagnosis and provision of effective treatment of the increasing number of patients with comorbidities [4,5]. Other prevalent changes include introducing emergency medicine as a separate specialty [6] and formalised use of triage systems [7]. Many different ways of organising the ED is evolving and the costs and effects are being debated [8]. A way of assessing the effect on the re-organisation and the many local initiatives is highly warranted.

Inspired by the private service sector’s way of monitoring and evaluating work processes, health care decision makers have seen the importance of adopting a similar view on management [8]. Hence, an increasing number of quality- and performance measurement initiatives have been integrated within the core operations. Performance measurement is a broad topic, which is rarely defined in detail. Most commonly, it is referred to as the process of quantifying actions, where measurement is a process of quantification and following action leads to performance [9]. Individual performance measures are defined as metrics that reflect effectiveness and/or efficiency of an action. A selection of such performance measures thus comprises of a performance measurement system which enables a more comprehensive evaluation of performance. Widely acknowledged performance measurement frameworks such as the Balanced Scorecard [10] and Business Excellence [11] have been implemented in health care to assure strategy alignment with operations. Even though a high percentage of performance measurements initiatives fail, mainly due to either being poorly designed or too difficult to implement in practice [12], successful implementation and use has been reported [13,14].

The fundamental idea of quality assurance in health care was originally to pass accreditations, whereas the healthcare sector now strives to converge quality improvements wherever possible. Many EDs have accepted the Institute of Medicine’s (IOM) report from 2001 called “Crossing the Quality Chasm” [15]. In this report, six quality domains are endorsed. These are *safety*, *effectiveness*, *patient*-*centeredness*, *timeliness*, *efficiency* and *fairness* (*equity*). The terms *efficiency* and *effectiveness* are often used interchangeably. Efficiency refers to the effectiveness of specific procedures whereas effectiveness regards the total outcome [15].

Different initiatives are continuously being presented in EDs in response to the IOM domains. In the United Kingdom (UK), crowded EDs were sought resolved by the introduction of the four hour target as a primary performance measure [16]. This means that only 98% of the patients may stay within the ED for more than four hours.

Focus on a single time-related measure does not necessarily correspond to high levels of quality and can potentially lead to dysfunctional behaviour [17]. Other important performance areas become unmonitored when focussing only on few ultimate measures. As an example, patients are without adequate treatment transferred to other wards more rapidly to keep length of stay in the ED within the accepted upper threshold limits. This can lead to reduced quality, increased costs and difficulties in retaining staff (sustainability). The outcome of the measure would be great yet the obtained quality would be poor.

Asking the clinicians in UK EDs about the subsequent effects of the four hour target resulted in a governmental report in which a total of eight performance measures to best represent quality were suggested by the Department of Health [18]. Eight performance measures were chosen on the basis of best possible evidence, formulated by lay-representatives and are weighted equally (in theory).

The UK EDs are not alone in the dilemma of determining how to evaluate new initiatives on key performance measures aligned with department visions. Similar problems such as crowding and scarce resources are struggled with elsewhere in the world. The selection of which performance measures to highlight also differs according to stakeholder perspective [19]. A clinician’s perspective on highly important performance measures is distinct compared to that of a patient, policy maker, or administrator, mainly due to the use of the measures for varying purposes. The entities may be subject to alteration over time depending on evolving clinical evidence, new practices and procedures, public opinions, and health system dynamics. Whereas a policy maker’s chief concern involves public accountability or a measurement framework reflecting ‘pay for performance’, the clinicians will demand procedural improvements for the benefit of enhanced treatment outcomes and clinical safety. From a patient’s perspective, the main focus will be on patient centeredness considered excellent medical treatment is delivered.

Consensus is still lacking on which measures are considered to be most accurate, extensive, clearly defined, and based on evidence [20,21]. Working towards a consensus of performance measures that reflect the general performance of an ED and whether or not certain quality improvement initiatives prove efficient is clearly warranted. A shared understanding of performance measures will enable continuous quality improvements and benchmarking opportunities both internally and externally over time.

The aim of this article is to present an overview of the highlighted performance measures suggested in internationally peer-reviewed review articles through the application of PRISMA guidelines.

## Methods

### Literature search strategy

This review gathers information on published results of review articles highlighting performance measures suitable for overall ED assessments. Identification of such articles was done through a systematic search in the databases of PubMed, Cochrane Library, and Web of Science conducted in the period of April to July, 2012. For all searches performed, the term “emergency department” or “ED” was used as fixed standard phrases. A selection of combined searches was conducted using the following text strings: emergency department, ED, performance indicator(s), performance measure(s), quality assessment, quality assurance, and quality improvement.

To investigate synonyms to the variable search terms, MeSH headings and wildcards were applied. The searches, with the variable search terms, and resulting number of hits are presented in Table 1.

**Table 1:** Search strings and resulting hits

**Search**	**Variable search string**	**# Hits**
1	performance measure/(performance measures)	13/(46)
2	performance indicator/(performance indicators)	13/(36)
3	quality measure/(quality measures)	24/(47)
4	quality indicator/(quality indicators)	19/(200)
5	quality assessment	67
6	quality evaluation	2
7	performance assessment	15
8	performance evaluation	13
9	quality assurance	657
10	quality improvement	9

PubMed differs between the singular and plural forms of phrases, hence the distinction shown in parenthesis. For example, the term “performance measures” is recognised as a different keyword compared to “performance measure” although nearly half of the articles reoccurred. A performed search is based on wordings in both titles and abstracts. The searches were performed separately using Boolean operators: “emergency department” OR “ED” AND the given search term. All search hits were filtered to only include reviews and structured reviews in terms of article type. The searches were performed according to the PRISMA guidelines [22].

### Inclusion/exclusion criteria

Articles were included in the systematic review if they managed to fulfil all of the following stated criteria: 1) the main purpose was to discuss, analyse, or promote performance measures that best reflect ED performance, 2) the articles were review articles, and 3) the articles reports macro-level performance measures, thus reflecting an overall departmental performance level.

Articles were excluded if 1) they referred to a specific patient group or illness, 2) the setting was different than EDs, 3) they did not touch upon performance measurement, 4) they investigated evidence behind selected indicators, 5) they described how measures were used or should be used in the future, 6) they directed criticism towards vaguely defined performance measures, and 7) the language was different from English.

### Selection

Selection of articles was performed independently by two of the authors (CMS and PJ) by reviewing titles and abstracts. If any doubts arose, the entire article was assessed. Afterwards, a decision about possible inclusion was made on the basis of a discussion between the two authors (CMS and PJ).

### Synthesis of results

According to the Traberg categorisation, all recommended performance measures can be allocated into the three categories; 1) patients, 2) employees, and 3) operations [23]. Traberg’s framework was chosen due to its sensible division of performance measures into clusters seen from a clinician’s viewpoint.

## Results

### Study selection

**Figure 1:**
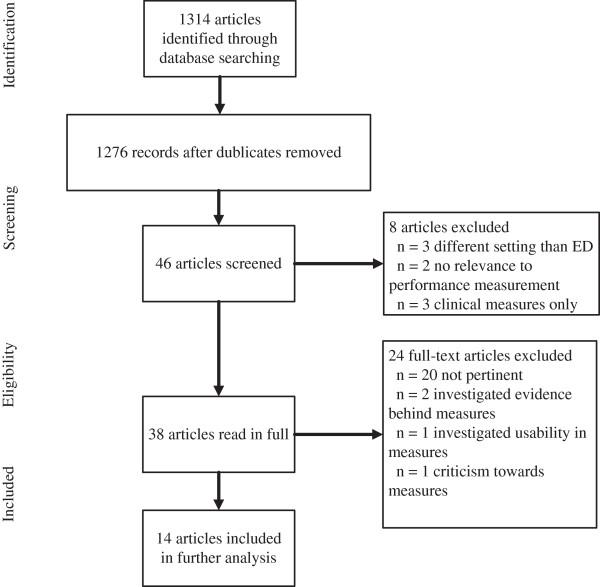
Flow-chart of article selection.

A total of 1314 titles were identified from the applied databases. 46 of these were unique. The unique titles were scanned on the basis of both title and abstract. Then, the inclusion/exclusion criteria were applied leaving 38 articles to be read in full extent. Of the 38 articles, 14 of these met the eligibility criteria and were included for further analysis. A flowchart presenting the article selection process is shown in Figure 1.

By accumulating all search hits presented in Table 1, 38 redundant articles were marked and excluded. A total of 1276 articles’ titles and abstracts were afterwards screened based on the inclusion/exclusion criteria. Any article title or abstract not deemed relevant by the authors were discarded (n = 1230). These articles were discarded due to 1) being conducted in a setting different from emergency departments or, 2) not relating to performance measurement, or 3) focusing on a specific clinical condition measure (for instance percentage of paediatric asthma patients prescribed anti-inflammatory medication or time to antibiotics given to patients suffering from pneumonia [8]).

The initial filtering returned a total of 46 articles in which all abstracts were read. Eight of these articles adopted an approach which was not in compliance with this study’s inclusion criteria. As an example of an excluded article, Persell et al., 2011 implemented and evaluated a quality improvement intervention, which included several clinical condition specific measures [24].

24 articles were not included in the final review due to 1) being non-pertinent, 2) investigated the evidence behind certain indicators [25,26], 3) described how measures were used and will be used in the future [27], or 4) directed criticism towards vaguely defined quality indicators [28]. 14 articles remained to be analysed and compared.

The reference lists of the 14 final articles were browsed for possible relevant articles, yet none met the criteria for inclusion.

### Characteristics of the included studies

A comparison of the included articles is presented in Table 2. The two last columns in Table 2 indicate first the preliminary pool of performance measures analysed and second the actual recommended performance measures.

**Table 2:** Presentation of included literature (* Focus indicates whether the suggested indicators are more generally applicable or refers to clinical conditions (e.g. indicators related to specific ailments))

**Corresponding author**	**Year**	**Objective**	Focus*	**Setting**	**Method**	***Gross indicatorportfolio***	***Recommended indicators***
McClelland et al. [33]	2012	Examination of practical aspects in collecting time-based ED measures	Time-relatedmeasures only (7)	American, EDs	Structured interviews and few data comparisons	7	7
Beniuk, Boyle & Clarkson [29]	2012	To prioritise quantified crowding measures to assess current ED status	Overall (8)	International EDs (USA, UK, Canada, Australia, Netherlands and Hong Kong)	Standard three round Delphi study	27	8
Alessandrini et al. [15]	2011	Proposition of a measurement framework specific for PEC practitioners and administrators	Overall (13) and condition specific (1)	American, PEDs	Point of departure in IOM recommendations. Alteration into Donabedian’s structure, process, outcome categorisation	120	14
Ekelund et al. [34]	2011	1) To assess feasibility in gathering benchmark data in Swedish EDs and 2) to evaluate patient throughput times and inflow patterns	Overall (4)	Sweden, EDs	Comparison of variables reflecting quality measures	4	4
Heyworth [35]	2011	1) Benefits and drawbacks associated with a single time-related measure and 2) proposed quality indicators to assess timeliness, quality, and safety	Overall (8)	United Kingdom, EDs	Description of current state in the UK; reflection on the quality indicators proposed by the Department of Health	8	8
Schull et al. [21]	2011	Seeks consensus on a set of parsimonious quality-of-care indicators for an ED	Overall (11) and condition specific (2)	Canada, EDs	Modified Delphi panel technique, three rounds	170	13
Welch et al. [32]	2011	Consensus of a standard set of performance measures in EDs related to patient flow	Overall (44)	American, North American Benchmark Summit (367 EDs)	Survey and audit	44	44
Coleman & Nicholl [16]	2010	Identification of a indicators usable for PCT commissioners and NHS decision makers to monitor performance	Overall (16)	United Kingdom, EDs and Urgent Care Units	Standard three round Delphi study	70	16
Hung & Chalut [30]	2008	1) Presents which indicators are deemed most useful to assess PEC and 2) which measures are currently being recorded	Overall (15)	Canada, PEDs	2-part questionnaire including a novel ranking formula to prioritize indicators	67	15
Guttmann et al. [31]	2006	Development of measures relevant for paediatric emergency care (children < 19)	Overall (6) and condition specific (8)	American, PEDs	Structured panel process with underlying literature review	109	14
Sibbritt, Isbister & Walker [36]	2006	Provision of a recommended list of performance indicators from routinely collected data in EDs	Overall (9)	Australia, EDs	Data collection and following SPC analysis	9	9
Solberg et al. [3]	2003	Identification of measures in EDs relevant for managing crowding	Overall (38)	American, EDs	Expert consensus on 113 measures; 10 investigators refined the measures to a total of 38	113	38
Graff et al. [8]	2002	How to critically evaluate quality in an ED	Overall (9) and condition specific (29)	American, EDs	Summary. Point of departure in IOM recommendations. Afterwards alteration into Donabedian’s structure, process, outcome categorisation	38	38
Lindsay et al. [20]	2002	A systematic approach to identify valid and relevant measures in an ED	Overall (8) and condition specific (13)	Canada, EDs	Modified Delphi panel technique, two rounds	104	21

*ED* Emergency Department, *IOM* Institute of Medicine, *NHS* National Health Services, *PCT* Primary Care Trust, *PEC* Paediatric Emergency Care, *PED* Paediatric Emergency Department, *SPC* Statistical Process Control.

No literature older than ten years that reviews overall ED performance measures was identified. The included articles formulate their primary objective differently but ultimately come up with a list of performance measures which reflect key performance- and quality-in-care measures in EDs. All these performance measures relate to a macro-level aspect, implying that these are generally applicable. In terms of the articles’ settings, USA and Canada have had the greatest focus on how to assess performance in EDs based on publications. The UK, Sweden and Australia have now also published their view on what performance measures to report. As units of analysis, paediatric EDs and general EDs were both eligible for analysis in this article since there is no difference in the generally applicable performance measures highlighted when referring to patient age. A differentiation between age and gender would be advisable if the performance measures were matched to specific clinical conditions.

With regards to the chosen approach, most of the articles apply a survey based approach consisting of two or more rounds of questioning panel members (commonly designated as the Delphi technique) [3,16,20,21,29-31]. This approach serves the purpose of finding consensus for a given topic by filtering responses through every stage. Two review papers report on interviews or audits [32-34]. A single article refers to a British governmental report [35]. Two articles elaborate on the IOM guidelines [8,15] and a single article includes performance measurement tracking by the application of statistical process control (SPC) [36].

A difference exists between the number of performance measures ultimately recommended and the gross pool of indicators investigated for several of the articles. These to amounts are listed as the two last columns of Table 2.

Duplicate performance measures were filtered out only if the wording differed slightly. The authors included core measures if the level of detail was deemed too specific. An example of a low abstraction level can be found in Welch et al. 2011 where the performance measure LOS is divided for admitted-, discharged-, observational-, and behavioural health patients [32].

All recommended performance measures are presented in Tables 3, 4 and 5 in compliance with Traberg’s three overall categories.

**Table 3:** Patient related measures

		**Alessandrini 2011**	**Beniuk 2012**	**Coleman 2010**	**Ekelund 2011**	**Guttmann 2006**	**Graff 2002**	**Heyworth 2011**	**Hung 2008**	**Lindsay 2002**	**McClelland 2012**	**Schull 2011**	**Sibbritt 2006**	**Solberg 2003**	**Welch 2011**
**Patients**															
Safety	Unintended incidents	x				x	x		x		x	x			
	Medication errors	x					x		x						
	Treatment errors						x					x			
	Missed diagnosis					x	x					x			
	Morbidity/mortality			x	x	x			x	x			x		
	Unplanned re-attendance (<72 hours)	x				x		x	x	x		x	x		
Patient centeredness	Complaints	x													x
	Patients Who Left Before Supposed To (PWLBST)														x
	LWBS (Left Without Being Seen)	x	x				x	x	x			x	x		x
	LBTC (Left Before Treatment Complete)		x											x	x
	LAMA (Left Against Medical Advice)														x
Satisfaction	Satisfaction (in general)/survey	x			x		x	x				x			

**Table 4:** Employee related performance measures

		**Alessandrini 2011**	**Beniuk 2012**	**Coleman 2010**	**Ekelund 2011**	**Guttmann 2006**	**Graff 2002**	**Heyworth 2011**	**Hung 2008**	**Lindsay 2002**	**McClelland 2012**	**Schull 2011**	**Sibbritt 2006**	**Solberg 2003**	**Welch 2011**
**Employees**															
Occupation profile	Educational positions												x		x
Work environment	Employee complaint ratio														x

**Table 5:** Operational performance measures; Note: double dashes between factors indicates a time interval

		**Alessandrini 2011**	**Beniuk 2012**	**Coleman 2010**	**Ekelund 2011**	**Guttmann 2006**	**Graff 2002**	**Heyworth 2011**	**Hung 2008**	**Lindsay 2002**	**McClelland 2012**	**Schull 2011**	**Sibbritt 2006**	**Solberg 2003**	**Welch 2011**
**Operations**															
Planning	Acute load													x	
	Bed occupancy rate												x	x	
	Boarding burden		x											x	
Utilization	Utilization rate (lab equipment)	x				x									
	Number of ECG’s taken									x					x
	Number of plain radiographic studies														x
	Number of CT studies														x
	Number of MRI studies														x
	Number of ultrasonic studies														x
	Number of laboratory studies														x
	Overall medication usage														x
	Number of behavioural health consultations														x
	Number of specialty consultations														x
	Utilization rate (employees)									x					
Efficiency	Throughput													x	
	ED admission transfer rate		x	x										x	
Time intervals	LOS (Length of Stay), total		x		x	x	x	x	x	x	x	x	x	x	x
	Ambulance off-loading time		x									x			x
	Arrival -- Registration														x
	Arrival -- Treatment space														x
	Arrival -- Clinical assessment	x	x		x		x	x	x			x			x
	Arrival -- Hospitalization	x		x					x			x		x	
	Arrival -- Init. triage	x	x										x		
	Arrival -- Init. treatment			x				x			x	x			
	Registration -- Init. triage	x							x						
	Registration -- Discharge/transfer								x						
	Triage -- Triage completed														x
	Triage -- Init. treatment	x							x				x		
	Admit decision -- Discharge													x	x
	Treatment space -- init. encounter														x
	Init. encounter -- Init.treatment			x											x
	Init. encounter -- Hospitalization											x			
	Init. encounter -- Clinical decision			x					x						
	Init. encounter -- Discharge/transfer											x			
	Disposition decision -- Discharge														x
	Hospitalization -- Discharge/transfer								x		x	x			
(diagnostic imaging)	Registration -- X-ray ordered														x
	X-ray ordered -- X-ray taken (radiology turnaround)						x				x			x	x
	Data ready -- Disposition decision time														x
(laboratory)	Blood sample ordered -- Blood sample result (lab turnaround)	x													x
(bed logistics)	Bed ordered -- Bed assigned														x

Some of the suggested performance measures connected to patients needs to be more precisely defined before use. Often indirect measures have to be used instead and that explains the common use of unplanned re-attendance as a performance measure, because it indirectly reflects a missed diagnosis or inadequate treatment. One may argue that the safety measures rather reflect operational efficiency. Such measures’ both quantitative and qualitative character explains why they are a part of the proposed framework. For instance, the number of unintended incidents is without value if not accompanied by a qualitative description to pinpoint what went wrong.

Most highlighted in the cluster *patient centeredness* was the outcome measure LWBS (patients who leave before being seen by a physician). This measure can be hypothesised to be related to patient satisfactory levels, as it is often related to crowding and extensive waiting times.

LWBS is also regarded as an important measure due to documented increased risks and adverse outcome in patients leaving before being treated [19,21,29].

A high rate in LWBS points toward potential systemic obstacles in patient reception or triage.

In terms of patient centeredness and satisfaction surveys, none of the included articles elaborates on which latent constructs must be recorded to reflect overall patient satisfaction levels. Much other literature addresses this issue using diverse approaches [37-39]. Employee related performance measures are presented in Table 4.

Only two performance measures connected to employees are suggested by Sibbritt et al. 2006 [36] and Welch et al. 2011 [32] (see Table 4). Insertion of the employee perspective in quality improvements has only recently been suggested by Crouch & Cooke in 2011 [40], entailing that in the future there may be a change in the demeanour of ED performance measurement. Employee related performance measures provide a pointer to which degree the current performance is sustainable. Sustainable performance is also linked to measures such as sickness absence rates, educational programme outcomes, and the amount of staff having the necessary competencies to fulfil their respective job descriptions [19]. In contrast to the employee related performance measures, operational performance measures have harvested more interest. These are presented in Table 5.

The operational performance measures deal primarily with effectiveness mainly related to time based registrations. Changes in working procedures serve a two-fold purpose; 1) timely treatment and 2) improving quality-in-care.

**Figure 2:**
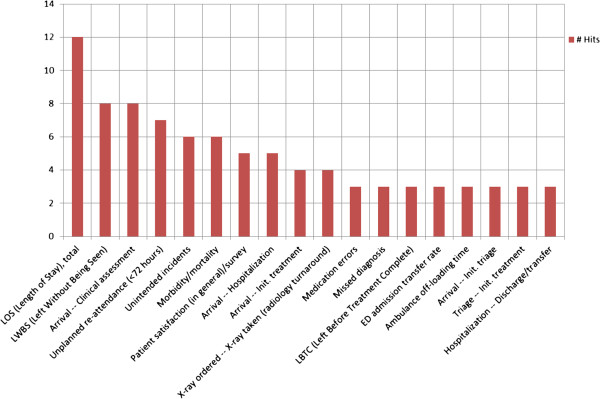
Top 25% of highlighted performance measures in included literature.

The main ED tasks are fast recognition and treatment of time-dependent critical conditions plus fast disposition to adequate level of care. Therefore, the great focus on time intervals is not a surprising result. LOS is far the most used time interval. LOS is an indirect overall measure of the efficiency of the whole ED stay. Keeping LOS short also means reducing crowding and keeping an efficient patient flow. Despite that timely treatment is one of the main performance goals for an ED, it is notable that time to treatment is only the ninth most highlighted performance measure (see Figure 2). LOS is often an easy parameter to retrieve from the ED computer system and is relatively easy to define. Time to treatment is more difficult to define and often not as easy to register in a standardised manner. In addition one could argue that time to treatment should be divided into treatment time related to triage category. Thus, data availability and easily defined measures could influence the choice of measures. Indeed, other stakeholders than clinicians contribute to the focus of timely treatment, especially in the wake of crowding. Such stakeholders are patient associations, politicians, and the media.

Presented in Figure 2 is the top 25% of the hits identified in the included literature.

## Discussion

The investigated articles differ in their approach, yet share their primary objective which is to analyse, discuss, or promote a series of performance measures that reflect key performance metrics and quality-in-care in emergency departments.

No literature older than ten years that reviews overall ED performance measures was found. During the recent five years, there has been an intensified debate on ED performance measurement. This comes in response to a previous low prioritisation of the emergency medicine area and an increase in ED patient volume over recent years.

A qualitative approach in choosing performance measures seems dominant. Especially the Delphi technique seeking consensus through either audits or questionnaires serves as a means to filter suggestions into core performance measures best suitable for ED assessments.

A total of 55 different performance measures are highlighted in the investigated literature. The level of abstraction in the included papers differs from four to 44 performance measures in total. Most of the suggested performance measures are independent on patient specific indicators and thus serve to reflect overall ED performance levels.

### Patients

Patient *safety* is challenging in the highly complex and time critical environment with undifferentiated patients in the ED. Thus, it is an absolutely essential measure which is confirmed by several recommended measures and is being suggested in most of the relevant literature. Tracking the conclusive outcome, mortality and morbidity, seems highly warranted but can be difficult to obtain, except for some of the well-developed countries that register much health statistics, for instance in Scandinavia. Especially mortality reviews engage clinicians and serve as a means for continuous quality improvements [19].

### Employees

As the most apparent stakeholder, the patient must remain as the paramount focus and all internal procedures must strive to yield as much value as possible to the quality-in-care. In the periphery of performance measurement focus, treatment services are performed by the employee, who is an essential resource for maintaining the daily operations. High quality treatment and optimal patient flow correlates with a high level of employee contentment, low turnover, and great seniority [41].

In the included literature, the employee aspect has to date not been given a high priority in the assessment of ED performance [21].

### Operations

Welch et al. raises the question of how and when to define that the actual patient progress has begun [32]. Does it start when the patient arrives at the ED or when the patient is registered in the ED administration system? Ideally, the registration begins when the patient enters the ED facility but in practice this is difficult to obtain. Therefore, the starting point often is at patient registration. Local circumstances from patient arrival to registration become a factor to include when benchmarking externally.

### How many performance measures to include?

Many emergency departments register large amounts of data. Probably, not all registered data is being used. As an ED decision-maker, it is impossible to investigate causes and effects from all registered data. Therefore, it is a necessity to determine which registrations appear most rich in information. It is important to find equilibrium of the required number of performance measures and invested work in collecting data. An extensive amount of performance measures may enable detailed analysis on the expense of extended time consumption. Few performance measures have the advantage of quick overview and thus lack the ability to take multiple aspects of performance into account. As can be read from Table 2, the amount of recommended performance measures varies greatly as a result of desired levels of detail.

### Criticism towards performance measurement in EDs

In parallel to the literature recommending certain performance measures, it is important to take notice of the literature which adopts a more critical perspective towards the focus on performance measures [26]. In this literature, evidence and formalisation of ED performance measures is questioned.

Evaluation of what is actually being measured, how to provide evidence for the choices of performance measures, and what the consequences are in implementation of any performance measurement framework is essential.

The authors acknowledge that once a set of performance measures is agreed upon, these should preferably be maintained over time to obtain sufficient data to add statistical strength, validity and reliability to each measure. It is then, ED decision-makers are provided the basis to decide whether to keep or discard given performance measures.

Sibbritt et al. suggests using statistical process control (SPC) when monitoring the department’s performance over time [36]. SPC is used to filter common cause variations from special cause variations. Application of SPC, either control- or run-charts, makes it possible to track alterations’ effects on key performance measures and is increasingly used in International Health Institute related projects [42].

### Data validity questionable

Gordon, Flottemesch, and Asplin report systematic errors and non-normal distributions in ED timestamps which weakens the foundation on which managers make their decisions [43]. Outcomes may also be prone to alterations if employees are given the opportunity to report better status than what is evident [44].

Once a set of performance measures are selected, validation should include a longitudinal study of the retained set of performance measures to ensure construct validity and that clinical processes are driven in the wanted direction [19].

### Perspective

Future challenges include a consensus on which performance measures should be in current focus to grasp crucial aspects of performance and contemporarily defining how these should be measured. Some performance measures may only be useful on a local level. However, comparing essential performance measures between EDs could promote learning that supports further quality improvements. It is imperative to agree upon definitions on key terms and measures to promote comparability between ED efficiency and effectiveness.

A joint set of identically defined performance measures across EDs would be beneficial in terms of benchmarking and ultimately continuous quality improvements.

Further studies should investigate the interconnectivity between the selected performance measures. Insight into the performance measures’ mutual impact allows for better understanding of ED performance. Furthermore, the use of SPC is deemed a highly important tool in data-driven leadership, since it is useful in measuring if initiatives have the intended effects or variations occur due to common causes.

## Conclusions

A structured literature review served the purpose of identifying which performance measures were analysed, discussed or recommended to assess ED performance on a macro-level. The most emphasised performance measures were time intervals and patient-related measures. Only few articles referred to the measurement of employee relevant measures.

In order to monitor the effect on different ED organisations and initiatives, consensus on a shared set of performance measures is needed. Consensus should include agreement on *how* and *when* the data registrations are gathered. These questions are crucial to address for streamlining performance measurement, which could allow for comparability between similar departments.

Moreover, investigation of the interconnectivity between the performance measures and how to measure if launched initiatives have the wanted effects is a sensible future research area.

## Competing interests

The authors have no competing interests to declare.

## Authors’ contributions

CMS has written the manuscript as well as conducted the literature review. PJ has critically reviewed the manuscript. JLF has formulated the study objective and critically reviewed the manuscript. All authors read and approved the final manuscript.
